# Developments for a New Spectral Irradiance Scale at the National Institute of Standards and Technology

**DOI:** 10.6028/jres.102.036

**Published:** 1997

**Authors:** Benjamin K. Tsai

**Affiliations:** National Institute of Standards and Technology, Gaithersburg, MD 20899-0001

**Keywords:** blackbody, filter radiometer, scale realization, spectral irradiance, uncertainty

## Abstract

Recent developments for a new spectral irradiance scale realization at the National Institute of Standards and Technology have been targeted to reduce the present relative expanded uncertainties of 0.67 % to 4.34 % (coverage factor of *k* = 2 and thus a 2 standard deviation estimate) in the spectral irradiance scale to 0.17 % for the range from 350 nm to 1100 nm. To accomplish this goal, a suite of filter radiometers calibrated using NIST’s high accuracy cryogenic radiometer have been used to measure the temperature of a high-temperature black-body. A comparison of the filter radiometer calibrations with the spectral irradiance scale along with an evaluation of the black-body calibration technique have been performed. With the aid of a monochromator, the calibrated filter radiometers will then be utilized to calibrate primary and secondary spectral irradiance standard lamps at NIST.

## 1. Introduction

A new method for an improved spectral irradiance scale is currently being developed at the National Institute of Standards and Technology. The goal of this project is to decrease the relative expanded uncertainty of the spectral irradiance scale in the 350 nm to 1100 nm region from the present 0.67 % to 4.34 % [1] to about 0.17 % using the proposed technique [2]. (Throughout this paper, all uncertainties are given as relative expanded uncertainties with a coverage factor of *k* = 2 and thus are two standard deviation estimates [3].) To accomplish this goal, several steps in the current measurement chain will be eliminated to reduce the uncertainty due to these steps. The NIST High Accuracy Cryogenic Radiometer (HACR), which has an uncertainty of 0.0266 % [4] at 633 nm, will serve as the primary standard instead of a gold-point blackbody, the temperature of which was determined from absolute spectral radiance measurements using absolute detectors and a room-temperature electrical substitution radiometer.

The present technique of realizing the spectral irradiance scale in the Facility for Automated Spectroradiometric Calibrations (FASCAL) [1] at NIST consists of several steps. First, the temperature of a variable temperature blackbody is determined by comparison with a gold-point blackbody. Next, based on the spectral radiance of the variable temperature blackbody, the spectral irradiance of a small integrating sphere source is calibrated and is transferred to the primary working irradiance lamps. Finally, test lamps are calibrated against the primary working standard lamps, which are recalibrated annually. A prism-grating monochromator is used in the measurement in each step. A complete description of the current spectral irradiance scale realization at NIST is described elsewhere [1].

The measurement technique that is now being developed involves three basic components: a high-temperature blackbody source, six filter radiometers, and a prism-grating monochromator. In the proposed spectral irradiance scale realization [2], filter radiometers will be used to determine the temperature or spectral radiance of a high temperature blackbody [5, 6]. By using a prism-grating monochromator equipped with irradiance collection optics, the spectral irradiance of the blackbody equipped with a limiting aperture of a known area will be used to determine the spectral irradiance of both the primary and test lamps. In this manner, the gold-point blackbody and integrating sphere source will be eliminated from the present calibration scheme, and the uncertainty in the spectral irradiance scale will be reduced through elimination of the most significant contributions from these two sources. These contributions include the uncertainties in the determination of the freezing temperature of gold and in transferring the radiance of the integrating sphere to the irradiance of the primary lamps.

A trap detector will be used as the transfer detector between the HACR and the filter radiometers. The absolute spectral responsivity of the trap detector will be determined by calibration with the HACR at a few wavelengths and fitting a model to the data over the whole spectrum of interest [7, 8]. Then, the trap detector will be assembled using a precision aperture with accurately known area and absolute spectral irradiance responsivity. Finally, the trap detector will be used to determine the absolute spectral responsivity of the six filter radiometers by using a monochromatic, Lambertian source currently being constructed at NIST, which provides the same geometrical and optical configurations as found in the proposed setup containing the high temperature blackbody and the filter radiometers.

In this paper a preliminary calibration of the filter radiometers using the existing measurement facility at NIST is described. The filter radiometers were characterized by measurements of the aperture area using a flux comparator technique and by calibration of the absolute spectral responsivity using the NIST Spectral Comparator Facility (SCF) [9, 10]. The filter radiometer calibrations were tested by comparing the predicted filter radiometer signals from a calibrated quart-halogen lamp with the corresponding measured signals. An evaluation of the methods used to calibrate the high-temperature blackbody source will be presented. Further testing of the measurement system involves using the monochromator and one or more filter radiometers to calibrate primary and secondary irradiance lamps for which the calibrations were already known.

## 2. Filter Radiometers

**Fig. 1:**
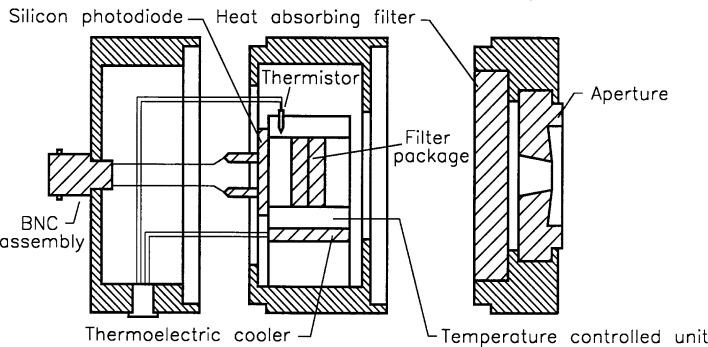
Schematic diagram of the filter radiometer.

Six filter radiometers, each composed of a silicon photodiode detector assembly, a narrow-band filter, and a precision aperture (Fig. 1), were assembled with various filters glued together and with heat absorbing filters where applicable. The filter and detector were calibrated as a unit using the NIST SCF, and the aperture areas were measured by flux comparison with a uniform source. These filter radiometers calibrated in the spectral range from 350 nm to 1100 nm were used to measure a known irradiance source. Two filter radiometers (F5, F6) have single heat-absorbing interference filters, while the second filter radiometer (F2) contains only a *V*(*λ*) filter, which is a filter that spectrally matches the CIE (*Commission Internationale de l’Eclairage*) spectral luminous efficiency function for photopic vision. The beryllium copper apertures were heat treated and polished to produce a sharp edge. To reduce stray radiation, these apertures were designed with sloped features on the front surface.

### 2.1 Calibration of Filter Radiometers

**Fig. 2:**
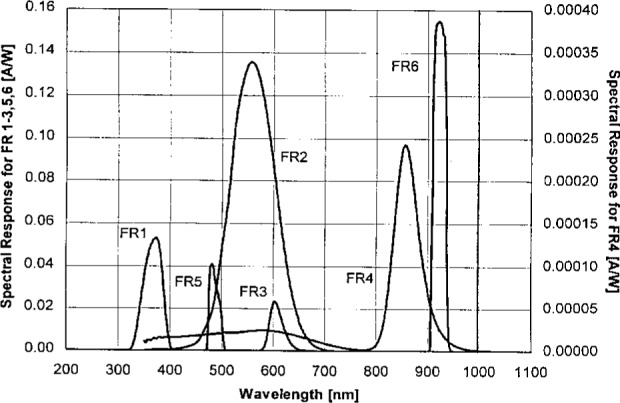
Graph of the absolute spectral responsivity for six filter radiometers, FR1 to FR6, measured on the Visible/Near Infrared (for FR2 to FR6) and the Ultraviolet (for FR1) Spectral Comparator Facilities.

The absolute spectral responsivity of the filter radiometers (FR) as a unit was determined by comparison with NIST standard detectors using the SCF, which is described in detail elsewhere [9, 10]. The areas of the apertures, which have a nominal diameter of 4 mm, were calculated using the ratio of the radiant flux from a uniform source through a test aperture to that through a standard aperture with known area. The uncertainty of this area measurement is approximately 0.07 % [11, 12]. Measurements of the absolute spectral responsivity curves for the six filter radiometers obtained from the NIST SCF setups were made in the 350 nm to 1100 nm region at 5 nm intervals with a monochromator bandwidth (full width at half maximum) of 4 nm (see Fig. 2). The band shape of the monochromator was roughly triangular [13]. The absolute spectral responsivity of FR4 has a peak response that is two orders of magnitude smaller than the other filter radiometers, and thus its data was plotted on the second ordinate on the right side. Since the responsivity in the visible region of FR4 seems to be significant, that portion must be measured more carefully or else be eliminated with a cutoff filter in the future. The absolute spectral responsivity curves of FR3, FR5, and FR6 have narrow bandwidths, but that of FR6 especially has high peak transmission and a narrow bandwidth. The broad bandwidth of FR2 is similar to that of a *V*(*λ*) filter. The absolute spectral responsivity curve of FR1 is dominant in the ultraviolet regime, while that of FR4 and FR6 peak in the near infrared region. The remainder have filters that transmit in the visible wavelengths.

The spatial response uniformity within the 2 mm diameter spot size at the silicon detector was measured with a 1.1 mm diameter beam at 0.5 mm increments. At 555 nm, the peak wavelength of FR2, the uniformity of FR2 was 0.06 %. Since the uniformity was acceptable, no correction for the spatial nonuniformity of the responsivity within the aperture was made.

### 2.2 Comparison of the Spectral Responsivity and the Spectral Irradiance Scales

As described in the introduction, the spectral irradiance scale at NIST is maintained in FASCAL [1]. A calibrated quartz-halogen standard irradiance lamp was used for this comparison. Measurements were made on FASCAL at selected wavelengths in the region from 350 nm to 1100 nm, and a blackbody function was used to interpolate for wavelengths between the calibrated points [1].

The statistic chosen for evaluation of the filter radiometer data is the difference expressed in percent,

$$\Delta_{r}=\frac{S_{p}-S_{m}}{S_{m}}\times 100,$$ (1)

where *S*_m_ and *S*_p_ are the measured and the predicted signals from the filter radiometer, respectively. In order to determine *S*_p_, an integral form of the measurement equation was applied,

$$S_{p}=DA\int_{\lambda }R(\lambda )E_{\lambda }(\lambda )d\lambda ,$$ (2)

where *R* is the absolute spectral responsivity of the filter radiometer as shown in Fig. 2, *D* is the amplifier gain, *A* is the area of the precision aperture on the front of each filter radiometer, *E_λ_* (*λ*) is the spectral irradiance of the quartz-halogen lamps used, and *λ* is wavelength. Simpson’s extended rule [14] was used to calculate the integral using a 5 nm step size.

The values of *S*_m_ and *S*_p_ are compared in Table 1. The first two columns designate the filter radiometer number and the wavelength of peak response, *λ*_max_. Next, the predicted signals from Eq. (2) and the measured signals are shown, followed by the difference in % as defined by Eq. (1). The last four columns in Table 1 state the estimated uncertainties for the amplifier gain, *D*, precision aperture area, the integral in Eq. (2), and the resulting uncertainty in the measured signal. The major contribution to the uncertainty of the signal comes from the uncertainty in the spectral irradiance of the standard lamps used. A bandwidth of 5 nm and a wavelength uncertainty of 0.1 nm were used to approximate the effect of the wavelength uncertainty [7], while the aperture area uncertainty was taken to be 0.035 % [11]. In general, the measured differences are within the estimated uncertainties. Using the filter radiometers, the quartz-halogen lamp signals can be predicted to within 0.7 %, as indicated in fifth column of Table 1. Half of the results are overestimated, while the remainder are underestimated, but there is no general pattern in the magnitudes of the differences. In general, there is good agreement between the measured and the predicted values.

## 3. High-Temperature Blackbody

A high-temperature blackbody is being developed at NIST for use in this project. The blackbody is a critical link in the spectral irradiance calibration chain. An understanding of its stability is required before any calibration is performed or measurements are made. The blackbody stability is inherently linked to the stability of the power supply and the control technique used. The blackbody control process involves an initial rough calibration of blackbody temperature versus current, from which a characteristic equation is developed. Computer calculations of required temperature versus actual temperature are then used to correct the current in order to control the blackbody temperature. The stability of the power supply to be used is found to be better than 0.05 % of full scale in 8 hours. This information is used to evaluate the most appropriate methods of blackbody stabilization. The full impact of this stability will be discussed in the conclusion section.

A thorough characterization of the high-temperature blackbody will include an assessment of the stability, repeatability, and uniformity of the spectral radiance, as well as measurements of the blackbody apparent emissivity, wall temperature, and cosine dependence of the spectral radiance. A proper feedback system is necessary to control the current within an acceptable limit. The uncertainty in the measured characteristics of the high-temperature blackbody influences the uncertainty in the spectral irradiance scale that is disseminated using the spectral irradiance standard lamps. The black-body characterization, the feedback control system, and the uncertainty analysis will not be described here but will be reported in the future. Characterization studies on similar high-temperature blackbody systems have been performed at the National Physical Laboratory [15] and at the Physikalisch-Technische Bundesanstalt [16].

### 3.1 Blackbody Description

**Fig. 3:**
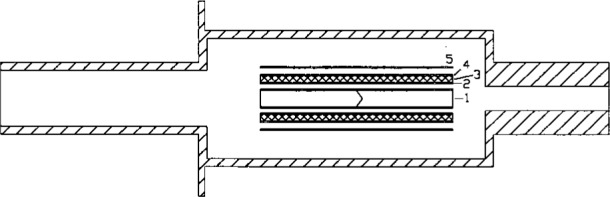
Simplified cross-sectional schematic of the high temperature graphite black body: 1—inner tube; 2—outer tube; 3—graphite cloth insulation; 4—insulation tube; 5—pyrolitic graphite shield.

The original high-temperature blackbody was first reported elsewhere [17], and a cross-sectional drawing is shown in Fig. 3. The blackbody inner tube is a 22 mm diameter cylindrical graphite cavity having an inverted cone in the center. A pyrolitic graphite disk is placed at each end of the cavity to help maintain temperature uniformity in the inner tube. Graphite cloth insulation tightly wrapped around an outer tube assists in maintaining good temperature uniformity in the blackbody cavity. An insulation tube and a pyrolitic graphite shield envelops the graphite cloth insulation. The cavity is heated resistively by conducting current through two graphite disks into the cavity, and the chamber is cooled with chilled water. For optimum performance, the chamber is first evacuated before the inner tube is purged with a moderate flow of argon gas to prevent contamination of the graphite with oxygen.

### 3.2 Blackbody Calibration

**Fig. 4:**
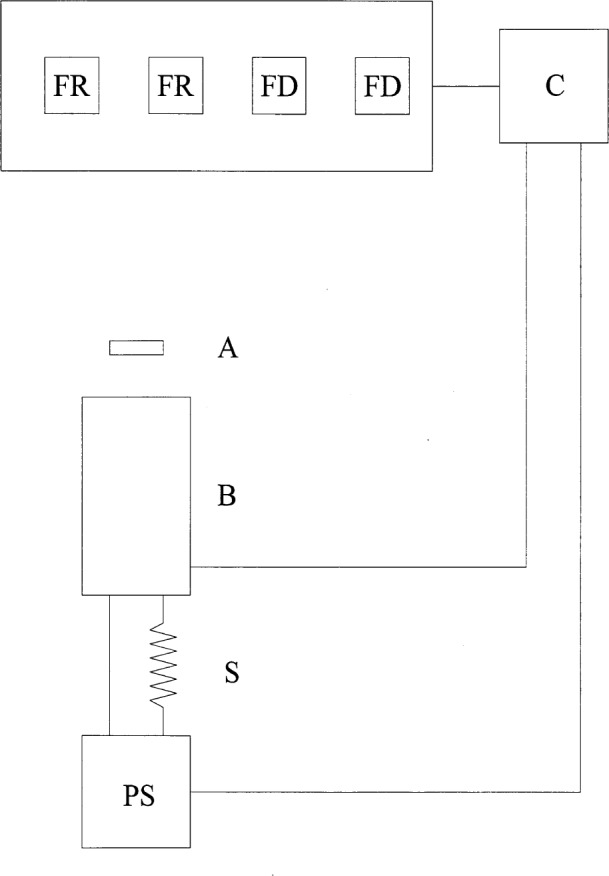
Schematic diagram of the calibration setup: A Precision aperture; B—high temperature blackbody; C—computer; F—filter radiometers; P—pyrometer; PS power supply; S—precision shunt.

The experimental layout for the calibration of the blackbody current versus temperature is shown in Fig. 4. The high-temperature blackbody source (B) has a precision aperture (A) a few centimeters in front of it, and flux through the aperture irradiates the detectors situated on a movable stage. The stage translates in a direction perpendicular to the optic axis, established by the blackbody and the detectors. Signals from the detectors, which include a single wavelength pyrometer (P) and filter radiometers (F), are received by a digital voltmeter and sent to a computer data acquisition system (C). The computer controls the stage translation and power supply current setting. The power supply (PS) controls the blackbody current, which is measured by a 300 A shunt (S) placed in series with the black-body. Initially, the blackbody is aligned relative to the filter radiometers and the pyrometer by using a beam-splitter-laser technique. The distance between the filter radiometers and the blackbody is measured with a distance gage having an internal micrometer. The water and the argon are turned on half an hour before heating the blackbody.

Mechanical positioning of each detector system (filter radiometer or pyrometer) is computer-controlled. A stage translates each detector to its previously aligned position. After all the detectors have taken data of the blackbody current and temperature, the computer sends the next current setting to the power supply. This is repeated until the final current setting is reached. The normal procedure consists of ramping the power supply current to a predetermined value for 5 min and then maintaining the current constant for 55 min. Using this procedure, the blackbody temperature is measured four times, twice with a pyrometer and twice with with FR2.

Determination of thetemperature of the blackbody is performed by using FR2, which contains a peak at 555 nm [17] and is calibrated for absolute spectral response *R*(*λ*) in the NIST SCF [10]. Calculations of the integral,

$$Q=\int_{\lambda }R(\lambda )\;L_{\lambda }(\lambda ,T)\;d\lambda ,$$ (3)

where *L* is the blackbody spectral radiance given by Planck’s equation at a wavelength and at a temperature *T*, were initially made for a selected number of temperatures. A new variable,

$$x=\log_{10}\;Q,$$ (4)

involving the common logarithm of *Q*, is used to handle the large dynamic range of *Q* values during the curve fitting process. In order to achieve an optimum combination of low χ^2^ and low number of fitting coefficients, a fifth degree polynomial fit for the temperature *T* is inferred from the expression

$$T=a_{0}+a_{1}x+a_{2}x^{2}+a_{3}x^{3}+a_{4}x^{4}+a_{5}x^{5}.$$ (5)

**Fig. 5:**
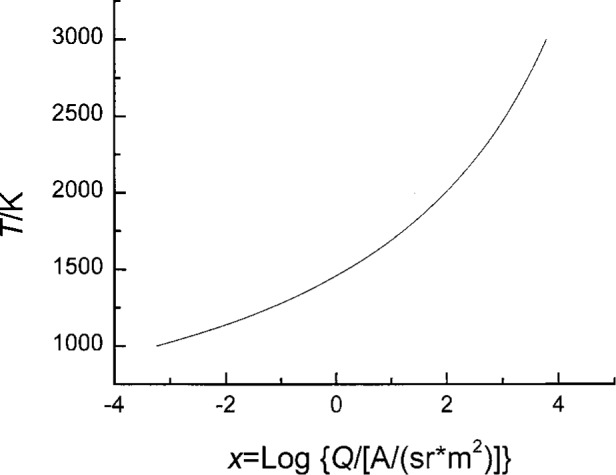
Graph of temperature as a function of *x* for the *V*(*λ*) filter radiometer after a fifth order polynomial curve fit has been performed. The equation for the curve is *T*/*K* = 1458.47507 + 203.77219 *x* + 26.44339 *x*^2^ + 2.88516 *x*^3^ + 0.6271 *x*^4^ + 0.13775 *x*^5^, where *x* = log_10_ {*Q*/[*A*/(sr · m^2^)]}.

Equations (3) through (5) are used to obtain the coefficients, *a_i_*, *i* = 1, 2,…, 5. The calibration curve for the V(*λ*) filter radiometer derived from the curve fit in Eq. (5) is plotted in Fig. 5. Since *Q* is related to the measured signal *S*, the amplifier gain *G*, and the geometrical factor *H* through the equation

$$Q=\frac{S}{GH}$$ (6)

**Fig. 6:**
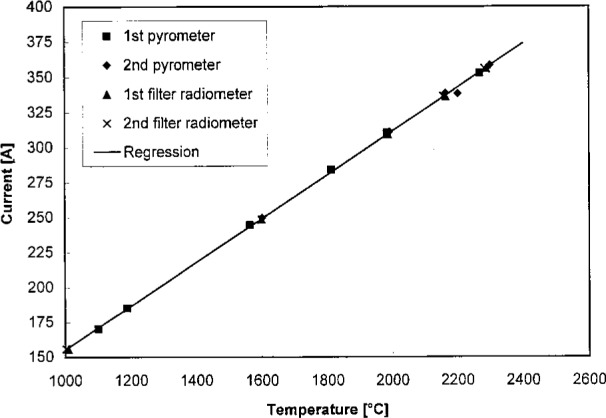
Four data sets showing the calibration of blackbody current vs. blackbody temperature as measured by a pyrometer or a filter radiometer. The equation of the fitted curve is *I*/*A* = 0.156 A · °C* *t*/°C – 0.544 A.

**Fig. 7:**
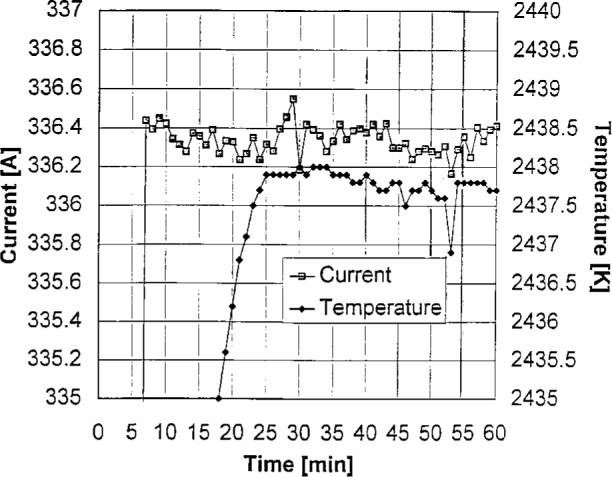
Graph of the high temperature blackbody temperature and current stability as a function of time at a nominal power supply current of 345 A.

Eqs. (4) through (6) allow the calculation of the temperature T from the signal S. Between 1273 K and 2573 K, the four data sets shown in Fig. 6 are fairly linear, displaying a slope of approximately 0.156 A/K or 6.41 K/A. Given a blackbody at a temperature of 2563 K and a current of 356 A, the uncertainties at 350 nm are shown in Table 2. The second and third columns show the experimental results and the values for reaching the final goal of 0.1 % uncertainty in relative spectral radiance, respectively. The thick horizontal line separates the two independent quantities above for the power supply from the three dependent quantities below for the blackbody. The goal of this project is to achieve 0.1 % uncertainty in the blackbody radiance. This means that the power supply needs to have the capability of controlling the blackbody current to within 0.007 %. In Fig. 7, the measured blackbody current stability for 30 min is 0.18 A, corresponding to a stability of 0.056 % of full scale. The realizable spectral radiance uncertainty is then about 0.72 %. Thus, the current stability needs to be improved by at least a factor of eight in order to achieve the 0.1 % uncertainty in the blackbody spectral radiance. The measured temperature stability is about 1.15 K, or about 0.045 % of full scale. The measurements are satisfactory for the rated performance of the power supply but are not acceptable for the final goals of this project.

As can be seen from Fig. 6, the temperature variations, which can arise from day-to-day variations or from hysteresis effects in a single calibration, can be as much as 35 K at any one current setting of the power supply. Without additional current control, daily calibrations of the blackbody must be made.

## 4. Future Work

The absolute spectral responsivity of the six filter radiometers in the spectral irradiance scale realization has been measured satisfactorily in the SCF at NIST. Utilizing the associated spectral responsivity curves to predict the signal from a calibrated quartz-halogen lamp and comparing this signal to the measured signal gave results within 0.7 %. This result tends to confirm the accuracy of both the spectral irradiance scale and the absolute spectral responsivity scales at NIST. A reduction of these differences can be made by calibrating the filter radiometers in the correct measurement geometry against the HACR using a trap detector with a precision aperture as the transfer standard.

**Fig. 8:**
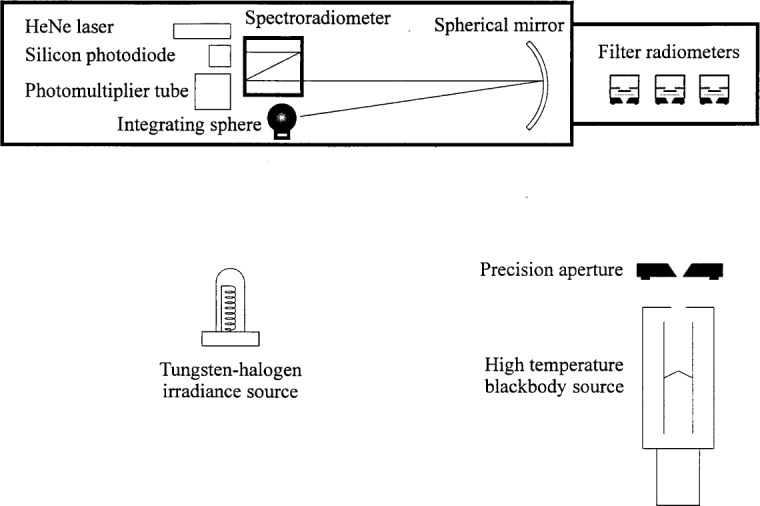
Schematic diagram of the setup for the spectral irradiance calibration of FEL lamps.

At the present time, the measured stability of the high temperature blackbody current is approximately 0.056 %. In order to achieve 0.1 % uncertainty in the blackbody radiance, the blackbody current must be controlled to better than 0.007 %. To improve the current stability and to decrease the long term current variations, a better power supply could be used or a feedback control method could be implemented. Because of their high cost, at present, we are investigating other methods of feedback control. One possible alternative that will be explored is to use a pyrometer in front of the high temperature blackbody with a beamsplitter or mirror. The mirror, which is oriented at 45° to the optic axis, reflects part of the blackbody beam to the pyrometer and can either have a hole in it or be placed on a rotating stage. Another option is to use a pyrometer in back of the high temperature blackbody to sight on the back of the inverted cone through the back hole for the feedback loop. In either the front or the back sighting configurations, the temperature is constantly monitored by the pyrometer, and the temperature variations are used to make the modifications to the power supply current. Using one of these feedback control systems, it will be necessary to minimize the current variations to less than 0.007 % in order to achieve the goal of measuring the blackbody spectral radiance with an uncertainty less than 0.1 %. The calibration of quartz-halogen lamps will be performed by the filter radiometer measurement of the blackbody temperature and by the comparison of the blackbody with the lamp using a spectroradiometer (Fig. 8). The calibrated filter radiometers will be used to measure the blackbody temperature and spectral radiance. Through a geometrical transfer, the spectral irradiance from the blackbody at the spectroradiometer can be determined. By calculation of the ratio between the signals from the blackbody and the lamp, the spectral irradiance of the lamp can be measured.

## Figures and Tables

**Table 1:** Calculation of quartz-halogen lamp signals measured by filter radiometers (FR)

FR No.	*λ*_max_/nm	*S*_p_/μA	*S*_m_/μA	Relative expanded uncertainties/%				
				*Δ*_r_/%	D	A	∫*RE*_λ_d*λ*	Total
FR1	365	7.0954 × 10^n3^	7.1103 × 10^−3^	− 0.21	0.006	0.07	1.88	1.88
FR2	555	4.1039 × 10^−1^	4.1267 × 10^−1^	− 0.55	0.006	0.07	1.06	1.06
FR3	605	2.6334 × 10^−2^	2.6427 × 10^−2^	− 0.35	0.006	0.07	1.85	1.85
FR4	881	1.2343 × 10^−3^	1.2336 × 10^−3^	+ 0.05	0.006	0.07	1.11	1.11
FR5	488	1.5372 × 10^−2^	1.5266 × 10^−2^	+ 0.69	0.006	0.07	2.43	2.43
FR6	920	2.4756 × 10^−1^	2.4619 × 10^−1^	+ 0.56	0.006	0.07	2.34	2.34

**Table 2:** Comparison of measured and target expanded uncertainties at 2563 K and 356 A

Uncertainty component	Expanded uncertainty	
	Measured	Target
Relative current stability	0.056 %	0.007 %
Current uncertainty	0.18 A	0.025 A
Temperature uncertainty	1.15 K	0.16 K
Relative temperature uncertainty	0.045 %	0.006 %
Relative radiance uncertainty	0.72 %	0.1 %
